# Glycine-Alanine Dipeptide Repeat Protein from C9-ALS Interacts with Sulfide Quinone Oxidoreductase (SQOR) to Induce the Activity of the NLRP3 Inflammasome in HMC3 Microglia: Irisflorentin Reverses This Interaction

**DOI:** 10.3390/antiox12101896

**Published:** 2023-10-23

**Authors:** Ru-Huei Fu, Hui-Jye Chen, Syuan-Yu Hong

**Affiliations:** 1 Graduate Institute of Biomedical Sciences, China Medical University, Taichung 40402, Taiwan; 2 Translational Medicine Research Center, China Medical University Hospital, Taichung 40447, Taiwan; 3 Ph.D. Program for Aging, China Medical University, Taichung 40402, Taiwan; 4 Department of Medicine, School of Medicine, China Medical University, Taichung 40447, Taiwan; 5 Division of Pediatric Neurology, China Medical University Children’s Hospital, Taichung 40447, Taiwan

**Keywords:** amyotrophic lateral sclerosis (ALS), C9ORF72, glycine-alanine dipeptide repeat proteins (GA-DPRs), NLRP3 inflammasome, sulfide quinone oxidoreductase (SQOR), ROS, mitochondria DNA, irisflorentin

## Abstract

Amyotrophic lateral sclerosis (ALS) is a fatal rare disease of progressive degeneration of motor neurons. The most common genetic mutation in ALS is the hexanucleotide repeat expansion (HRE) located in the first intron of the C9orf72 gene (C9-ALS). HRE can produce dipeptide repeat proteins (DPRs) such as poly glycine-alanine (GA) in a repeat-associated non-ATG (RAN) translation. GA-DPR has been shown to be toxic to motor neurons in various biological models. However, its effects on microglia involved in C9-ALS have not been reported. Here, we show that GA-DPR (GA_50_) activates the NLR family pyrin domain containing 3 (NLRP3) inflammasome in a human HMC3 microglia model. MCC950 (specific inhibitor of the NLRP3) treatment can abrogate this activity. Next, using yeast two-hybrid screening, we identified sulfide quinone oxidoreductase (SQOR) as a GA_50_ interacting protein. SQOR knockdown in HMC3 cells can significantly induce the activity of the NLRP3 inflammasome by upregulating the level of intracellular reactive oxygen species and the cytoplasmic escape of mitochondrial DNA. Furthermore, we obtained irisflorentin as an effective blocker of the interaction between SQOR and GA_50_, thus inhibiting NLRP3 inflammasome activity in GA_50_-expressing HMC3 cells. These results imply the association of GA-DPR, SQOR, and NLRP3 inflammasomes in microglia and establish a treatment strategy for C9-ALS with irisflorentin.

## 1. Introduction

Amyotrophic lateral sclerosis (ALS), a motor neuron disease (MND), causes progressive degeneration of the upper and lower motor neurons in the brain and spinal cord. Muscle stiffness, twitching, and atrophy are the characteristics of this disease. Progressive deterioration of speaking and swallowing ability eventually leads to respiratory failure. The average survival period of ALS from onset to death is approximately 3–5 years. The disease affects about 7.7 out of every 100,000 Americans each year and is most common among white men aged 60–69 [1]. At present, there is no effective method to delay the establishment of the disease and cure ALS. Although the drug riluzole has been approved, it only extends life by about two to three months and does not reverse the activity of damaged motor neurons [2].

ALS has an unknown cause in 90–95% of cases (sporadic ALS). Approximately 5–10% of cases are genetically related (familial ALS). To date, many genetic mutations associated with ALS have been identified, including *C9orf72*, *SOD1*, *FUS*, *TARDBP/TDP-43*, *ANG*, *SETX*, *ALS2*, and *VAPB*. These genetic variants also contribute to the development of sporadic ALS [3,4]. *C9orf72* is the most common genetic variant (40%, familial ALS) and occurs in a high proportion (8–10%) of sporadic ALS patients [5]. This gene is highly expressed in ALS-associated motor neuron populations [6,7]. In a normal person, intron one of *C9orf72* contains, at most, 20–30 hexanucleotide repeats (GGGGCC; G_4_C_2_), but, in people with *C9orf72*-associated ALS (C9-ALS), this number can expand to hundreds to several thousand repeats, which is called hexanucleotide repeat expansion (HRE) [8,9].

The mechanisms by which HREs cause disease are still poorly understood. The loss of function (haploinsufficiency) and gain of function (either toxic RNA or protein products) in C9-ALS molecular pathology have been confirmed by related studies [10,11]. C9ORF72 protein is mainly distributed in the cytoplasm or synapses of cerebral cortex neurons and brain/spinal cord motor neurons [12]. Its loss may be related to the defect of endosomal trafficking, autophagy [13], axonal synapse, suppression of inflammation and autoimmunity [14], translation, unfolded protein response, and stress granule (SG) formation [15]. In the gain of function, aberrant RNAs containing extended HREs can block many functions in the nucleus [16] or be translated by an unconventional method called the repeat-associated non-ATG mechanism (RAN) [17,18,19,20]. This resulted in five dipeptide repeat proteins (DPR) (Gly-Ala, Gly-Pro, Gly-Arg, Pro-Ala, and Pro-Arg) from ribosomal reading frames of sense or antisense directions generated and accumulated in the central nervous system of patients with C9-ALS [21]. However, studies have shown that only poly-GA has the highest expression in the brains of patients with C9-ALS [22], and its distribution is very similar to the neuronal cytoplasmic insoluble and ubiquitinated p62-positive inclusions usually observed in patients [23].

GA-DPR is cytotoxic and causes dysfunction of the ubiquitin–proteasome system [24], caspase-3 activation (apoptosis), damage of neurite outgrowth, endoplasmic reticulum stress [25], nuclear membrane destruction, obstruction of nucleocytoplasmic transport [26], and TDP-43 cleavage [27]/aggregation [28] in neuronal cells. GA-DPR also depletes the level of soluble Unc119, inhibits dendritic arborization, and leads to apoptosis of primary neurons [29]. Other studies have shown that if GA aggregates exist in neurites in mouse models and patient-induced pluripotent stem cell lines, they will reduce synaptic vesicle-associated protein 2 (SV2) levels and affect Ca^2+^influx and synaptic vesicle release, ultimately affecting synaptic function [22]. In *C. elegans* models, GA-DPR induces neurodegeneration, leading to movement impairments. Simultaneously exhibits longevity defects [30].

Current research shows that C9-ALS may abnormally activate the immune system [31]. Gut bacteria-induced systemic and neuroinflammation mediated by TMEM173-induced type I interferon has been observed in C9-ALS mice [32]. Synthesis of pro-inflammatory chemokines and cytokines was also found to be upregulated in the CSF and serum of C9-ALS patients [33]. Recent evidence revealed that, in addition to motor neurons, microglia may also be the primary cell responsible for ALS [34]. Furthermore, interferon-responsive proinflammatory microglial activation was characterized by transcriptome analysis in GA-DPR mice and C9-ALS patients [35]. Notably, NLRP3 (NOD-, LRR-, and pyrin domain 3) inflammasome activation in microglia is frequently observed in ALS-associated neuroinflammation in patient and mouse models [36]. It induces the activation of caspase 1 and then promotes the maturation and release of the pro-inflammatory cytokines IL-1β and IL-18, as well as activating gasdermin D-dependent pyroptosis [37]. Here, we were interested in whether *C9orf72*-associated GA-DPR stimulated microglial NLRP3 inflammasome activity, leading to ALS symptoms. Our results showed that endogenous GA_50_ can effectively cause NLRP3 inflammasome activation in HMC3 cells (human microglial cell line). In addition, we also confirmed that the activity induction was partly caused by the interaction affecting the function of sulfide quinone oxidoreductase (SQOR, GenBank: NM_001271213) protein, resulting in mitochondrial ROS generation and cytoplasmic escape of mitochondrial DNA. Finally, we found that the favonoid “irisflorentin” from *Belamcanda chinensis* (L.) DC could block the interaction between GA-DPR and SQOR and showed the potential to ameliorate C9-ALS-associated GA-DPR toxicity in cell models. Irisflorentin has the opportunity to be a potential option for the treatment of C9-ALS.

## 2. Materials and Methods

### 2.1. Chemicals and Maintenance of HMC3 Cell Lines

Chemicals used in this study were purchased from Sigma-Aldrich (St. Louis, MO, USA) unless otherwise stated. Reagents and media were acquired from Gibco (Thermo Fisher Scientific, Waltham, MA, USA). HMC3 human microglia were provided by Shao-Chih Chiu (China Medical University, Taichung, Taiwan). Cell cultures were performed as described in previous publications [38].

### 2.2. Construction and Transfection of Plasmid

Coding DNA for GA_50_ and SQOR was synthesized by Genewiz (South Plainfield, NJ, USA). The DNA of GA_50_ was cloned into the pcDNA 3.1/myc-His vector (Invitrogen, Waltham, MA, USA), pGBKT7 vector (Clontech, Mountain View, CA, USA), pGADT7 vector (Clontech), pCMV -Myc (Clontech), or pCMV-HA (Clontech). The SQOR fragment was inserted into pGBKT7, pGADT7, pCMV-Myc, or pCMV-HA vectors. Plasmids were transfected into HMC3 or 293T cell lines using Lipofectamine 2000 reagent (Invitrogen) according to the manufacturer’s protocols. Transfectants were selected using G418 (1.5 mg/mL). Yeast transformation for the pGBKT7 or pGADT7 plasmid was performed using Quick and Easy Transformation Mix (Clontech) according to the manufacturer’s protocol.

### 2.3. Immunofluorescence Analysis of HMC3 Cells

Immunofluorescence staining was performed according to our previously published protocol [38]. In short, cells adhered to the coverslip were fixed using paraformaldehyde (4%), followed by permeation with Triton X-100 (0.2%). The coverslips were then blocked in a milk solution, and then primary antibody was added to swing overnight (4 °C). The next day, the washed cells were added to Alexa Fluor 488-linked anti-mouse and Alexa Fluor 568-linked anti-rabbit secondary antibodies (both from goat, Invitrogen) at 25 °C for 1 h. Afterwards, cells were washed, and DAPI was used to stain nuclei. Finally, the fluorescence signal was observed using a fluorescence microscope. Myc, SQOR, and NF-κB p65 antibodies were obtained from Cell Signaling Technology (Beverly, MA, USA).

### 2.4. Acquirements of Nuclear Extracts and Transcription Factor Assay of NF-κB p65 in HMC3 Cells

We used the nuclear extraction kit (Sigma-Aldrich) to extract proteins in the nucleus according to the manufacturer’s manual. For the activity assay of NFκBp65, we employed the NFκB p65 EZ-TFA Transcription Factor Assay Colorimetric Kit purchased form Millipore (Temecula, CA, USA) according to the instruction manual.

### 2.5. Protein Extraction and Western Blot Analysis of HMC3 Cells

Protein extraction and Western blotting were performed using the experimental method described in the previous literature [38]. In summary, we added RIPA buffer (Millipore) containing aprotinin, leupeptin, PMSF, and phosphatase inhibitors to culture cells. The cells were then scraped, and cell lysates were collected. For Western blotting, we loaded cell lysates (50 µg) onto 7.5–12.5% SDS-PAGE for analysis. The location and signal intensity of specific proteins in PVDF membranes were determined using an ECL reagent (Amersham Biosciences, Piscataway, NJ, USA) and a luminescence imaging sensor (UVP, Upland, CA, USA). Primary antibodies, including NF-κB p65, NLPR3, ASC, caspase 1, IL-1β, IL-18, and GAPDH, were obtained from Cell Signaling Technology. HRP-linked anti-rabbit and anti-mouse secondary antibodies (both from goat) were acquired from PerkinElmer, Inc. (Boston, MA, USA).

### 2.6. Analysis of IL-1β and IL-18 in the Conditioned Media of HMC3 Cells

Conditioned medium from HMC3 cells was centrifuged at low speed (1000× *g*, 5 min), and the supernatant was collected and passed through a filter tip (0.22 µm) to remove cell debris. Next, IL-1β, IL-18, and c-Myc ELISA were performed according to the manufacturer’s instructions (invitrogen). Each sample was tested with 50 µL of conditioned medium and quantified on a microplate reader.

### 2.7. In Situ TUNEL Analysis of HMC3 Cells

The number of HMC3 cells exhibiting apoptosis in situ was counted using the Click-iT™ Plus TUNEL Assay Kit (invitrogen) according to the manufacturer’s instructions. Briefly, after fixation (4% paraformaldehyde) and permeabilization (0.25% Triton™ X-100/PBS), cells on coverslips were incubated with Tdt reaction mixture for 60 min. Next, Click-iT™ plus TUNEL reaction mix (containing Alexa Fluor™ 488 dye) was added to cells for 30 min in the dark. Finally, the number of apoptotic cells was observed and counted with a fluorescent microscope.

### 2.8. Detection of Apoptosis in HMC3 Cells by Flow Cytometry

Apoptosis analysis was performed using the FITC Annexin-V Apoptosis Detection Kit I (BD Biosciences Pharmingen, San Diego, CA, USA) according to the manufacturer’s protocol. Briefly, we added 100 μL of binding buffer to cells harvested after washing. Next, cells were stained with annexin-V FITC and PI for 15 min in the dark. Then, 400 μL of binding buffer was added, and the ratio of apoptotic cells was immediately determined using a flow cytometer. The gate for cell collection was set to contain 10,000 events per sample.

### 2.9. HMC3 Cells Were Treated with Inhibitor of NLRP3 Inflammasome

MCC950, a dairylsulfonylurea compound, is a potent and selective inhibitor of the NLRP3 inflammasome. We incubated GA_50_-transfected HMC3 cells in fresh medium containing MCC950 for 24 h prior to analysis.

### 2.10. Using a Yeast Two-Hybrid Library to Screen GA-DPR-Interacting Proteins

The Matchmaker cDNA library construction of HMC3 cells and the two-hybrid assay of Matchmaker GAL4 were performed according to the operation manual provided by the manufacturer (Clontech). Briefly, we transformed the pGBKT7/GA_50_ plasmid into the AH109 strain of *Saccharomyces cerevisiae* and the HMC3 cell cDNA library constructed in the pGADT7 vector into the Y187 strain. Next, the bait train and library strains were mated to generate diploids. Finally, the interaction of GA_50_ with candidate proteins can be confirmed by the positive clones on SD/-Ade/-His/-Leu/-Trp X-α-gal plates. In addition, we recovered the pGADT7 plasmid from positive yeast clones using a yeast plasmid miniprep I kit from Zymo Research Corporation (Irvine, CA, USA) and sequenced it to identify the interaction genes.

### 2.11. Yeast Two-Hybrid Assay of GA_50_ and SQOR

We subcloned GA_50_ and SQOR DNA fragments into pGBKT7 or pGADT7 vectors. Next, pGBKT7 or pGADT7 plasmids were transformed into AH109 and Y187 yeast strains. Finally, we performed a two-hybrid assay of Matchmaker GAL4, the procedure of which is described in Section 2.10.

### 2.12. Co-Immunoprecipitation Analysis of GA_50_ and SQOR

We refer to the experimental procedures described in the previously published literature for co-immunoprecipitation analysis [38]. First, the pCMV/GA_50_/Myc plasmid and pCMV/SQOR/HA plasmid were co-transfected into 293 T cells according to the transfection method described in Section 2.2. After 24 h, cell lysates were collected using Pierce™ IP Lysis Buffer (Thermo Fisher Scientific). Next, supernatants were immunoprecipitated with anti-Myc-Tag antibody or normal immunoglobulin G from rabbits for 2 h (4 °C). Beads of Protein G-Sepharose were then added for 1 h. Finally, the co-immunoprecipitation complexes were washed and identified by Western blotting using mouse anti-HA-Tag monoclonal antibody (Cell Signaling Technology). In addition, we performed immunoprecipitation of cell lysates with mouse anti-HA-tag monoclonal antibody and Western blotting with rabbit anti-Myc-tag monoclonal antibody. In another set of experiments, we co-transfected pCMV/SQOR/Myc plasmids and pCMV/GA50/HA plasmids into 293T cells and performed co-immunoprecipitation as described above.

### 2.13. siRNA Treatment of SQOR in HMC3 Cells

HMC3 cells were seeded on culture plates (6-well, 2.0 × 10^5^ cells/well) and grown to 70% confluence. SQOR siRNA (75 nM) or non-targeting control siRNA were then delivered into cells using Lipofectamine 2000 Reagent (Invitrogen) according to the producer’s protocol. After 24 h, cells and media were harvested for subsequent experiments. SQOR siRNA was purchased from Sigma-Aldrich and its product number is EHU032561.

### 2.14. Reactive Oxygen Species (ROS) Assay in HMC3 Cells

We measured intracellular ROS levels using a permeable H2DCFDA (DCFH-DA) (Sigma-Aldrich) probe as a marker according to a method previously described [39]. The fluorescence intensity of the probe after cell staining can be observed by fluorescence microscopy and detected by flow cytometry.

### 2.15. Measurement and Quantification of Cytoplasmic mtDNA in HMC3 Cells

We measured cytoplasmic mtDNA by general PCR or RT-qPCR analysis according to the experimental procedures described in the published literature [39]. In brief, half of the cells in each group were lysed with mild lysis buffer (containing 0.1% NP-40) and the other half with strong lysis buffer (containing 0.1% NP-40 and 0.1% SDS). Cells were treated with lysis buffer on ice and centrifuged at 4 °C. Next, the supernatant was passed through a Genomic DNA isolation kit (Sigma-Aldrich) to purify cytoplasmic mtDNA and total cellular DNA. Finally, we used PCR and RT-qPCR for analysis. The ratio of mtDNA to total DNA reflects the degree of cytoplasmic DNA escape.

### 2.16. Irisflorentin Treatment and Growth Assay of a Yeast Two-Hybrid Based on the Interaction between GA_50_ and SQOR

Synthetic irisflorentin (IFT, mol. wt. 386.35, 98% purity) was prepared as a 100 mM stock solution (in DMSO) and stored at −20 °C. Diploid yeast carrying BD-/AD-, BD-p53/AD-T, BD-GA_50_/AD-S3 (SQOR), or BD-S3/AD-GA_50_, respectively, were cultured in SD/-Leu/-Trp broth containing serially diluted IFT overnight until they reached the log or mid-log phase (30 °C). In the yeast spot assay, we normalized the diploid cultures. Next, serial dilutions were made and spotted (10 μL) onto non-selective (SD/-Leu/-Trp) or selective (SD/-Ade/-His/-Leu/-Trp) dishes and grown at 30 °C for 3 days. In absorbance assay experiments, diploid cultures from each group were normalized and cultured in non-selective or selective broth with serial dilution IFT. OD values were recorded every 12 h during the 48 h experiment.

### 2.17. Toxicity Analysis and Treatment of Irisflorentin on HMC3 Cells

The toxicity of irisflorentin to HMC3 cells was confirmed using an MTT cell survival assay. We treated HMC3 cells for 24 h with serial dilutions of IFT. The cells were then replaced with fresh medium and incubated with MTT (5 mg/mL) at 37 °C for 2 h. Finally, the cells were washed with isopropanol. Cell viability was quantified using a spectrophotometer. On IFT treatment, we replaced GA50-expressing HMC3 cells with fresh medium and treated them with IFT for 24 h, then collected cells and conditioned medium for subsequent experiments.

### 2.18. Statistical Work on the Data for This Study

Each work in this study was implemented in triplicate. We used SAS software 9.3 (SAS, Institute. Inc., Cary, NC, USA) for general statistical analysis of the data of this study. Study data are presented as mean ± standard deviation (SD). Furthermore, statistical significance was determined by using one-way analysis of variance (ANOVA) and Tukey’s test. Again, comparisons between two groups were performed using Student’s *t*-test. Statistical significance is indicated with a *p*-value < 0.05.

## 3. Results

### 3.1. Expression of Glycine-Alanine-Dipeptide Repeat Protein (GA-DPR) Causes NLRP3 Inflammasome Activity in a Human HMC3 Microglia Cell Model

**Figure 1:**
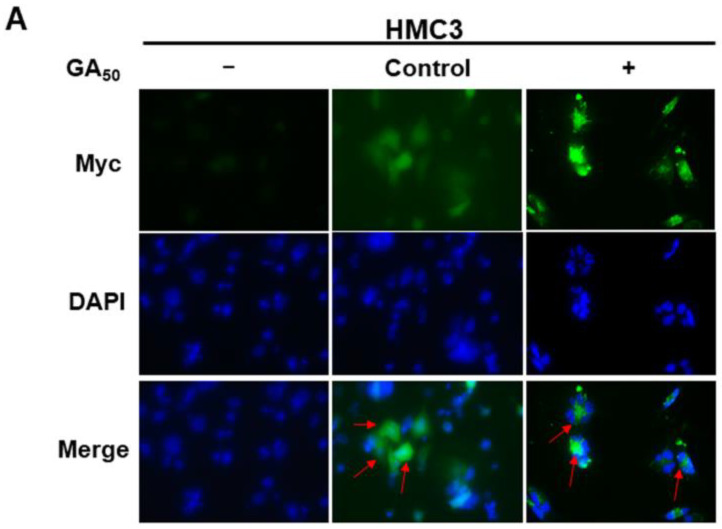

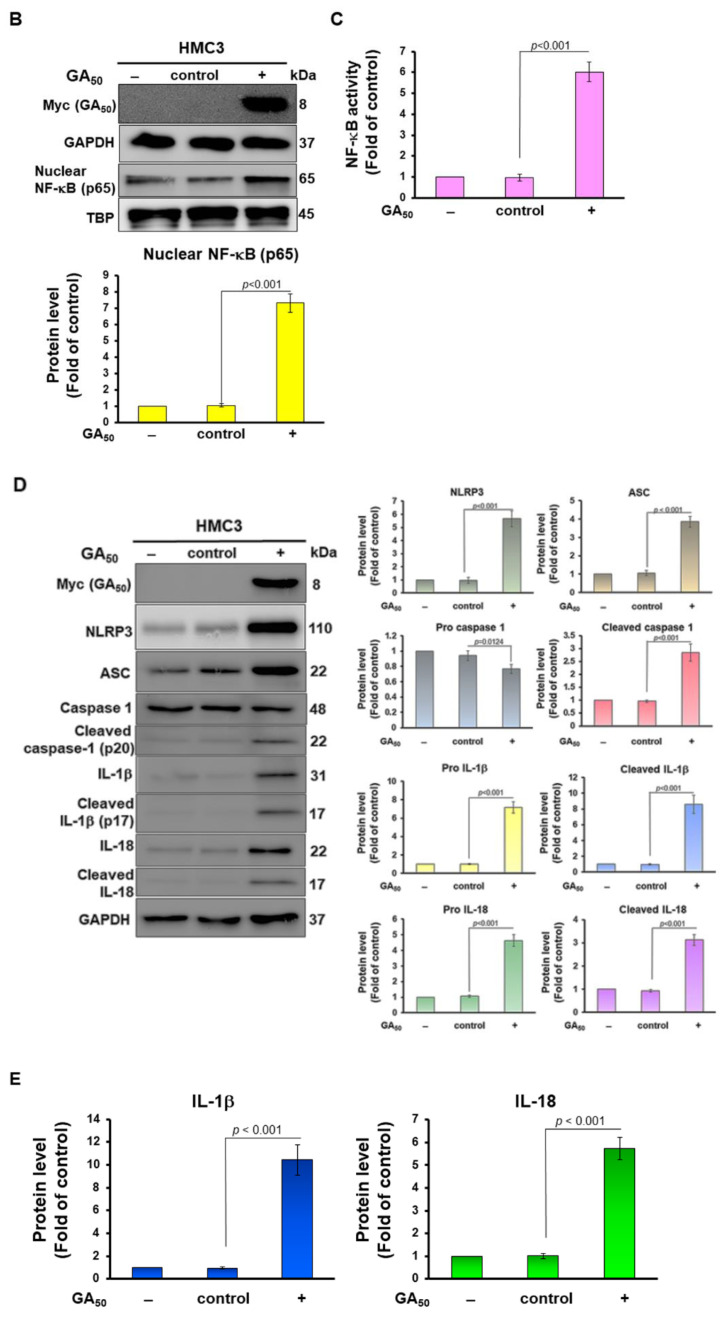

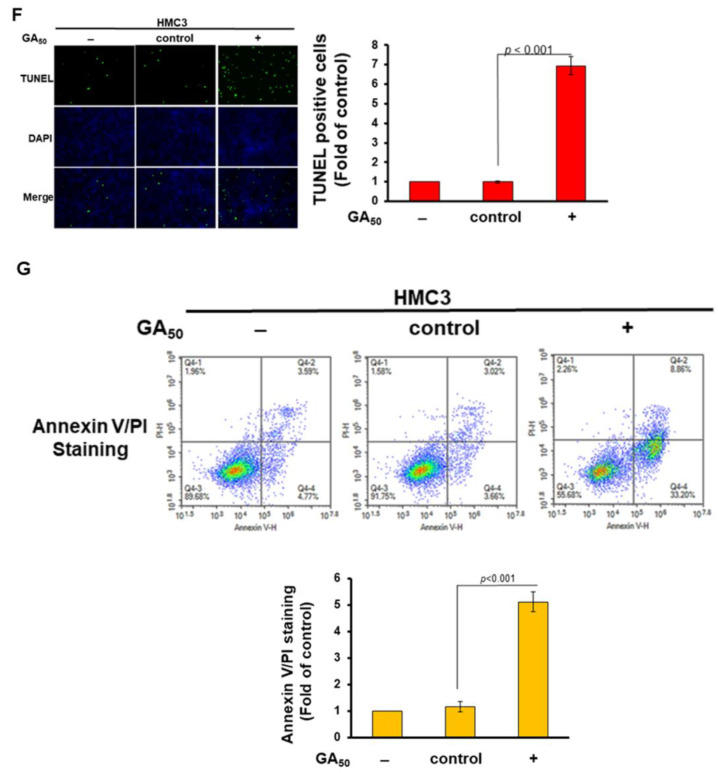
Intracellular expression of GA50 in human HMC3 microglia leads to NLRP3 inflammasome activation. The control vector or GA_50_ plasmid was transfected into HMC3 cells for 24 h. (**A**) Immunofluorescence staining of GA_50_ using anti-Myc-tag antibody (green). The location of the nuclei was confirmed by DAPI staining. The magnification of a microscope is 400X. GA_50_ mainly forms aggregates in the cytoplasm (red arrows). (**B**) The amount of NF-κB p65 in the nucleus was evaluated by Western blotting and quantified using ImageJ software (version 1.53). TBP protein is an internal loading control. (**C**) ELISA analysis quantifies the transcriptional activity of NF-κB p65. (**D**) Expression of GA_50_ (Myc), NLRP3, ASC, caspase-1, IL-1β, and IL-18 in cell lysates was analyzed by Western blot and quantified using ImageJ software (version 1.53). GAPDH was employed as an internal loading control. (**E**) The amount of IL-1β and IL-18 secreted in conditional media was determined using ELISA assay. (**F**) The number of cells with chromosomal breaks was estimated using TUNEL assay. The magnification of a microscope is 100X. (**G**) Ratios of pyroptosis cell were determined by flow cytometry with annexin v-FITC and propidium iodide (PI) staining. The histogram shows the pyroptosis rate [(Q1 + Q2)/(Q1 + Q2 + Q3 + Q4) × 100%].

The mechanism of the NLRP3 inflammasome involves the activation of NF-κB and the formation of mature caspase-1, mature IL-1β, and mature IL-18 (mIL-18) [32]. First, we investigated whether glycine-alanine dipeptide repeat protein (GA-DPR) could cause NLRP3 inflammasome activity in microglia. We expressed GA-DPR (GA_50_) by transient transfection in a human HMC3 microglial cell line. After 24 h of transfection by immunofluorescence analysis, we found that GA50 was mainly distributed in the cytoplasm to form aggregates (Figure 1A). In addition, Western blot analysis showed that the expression of GA_50_ increased the amount of NF-κB p65 in the nucleus (*p* < 0.001, Figure 1B). Likewise, ELISA analysis of transcription factors showed (*p* < 0.001, Figure 1C) that expression of GA_50_ could significantly induce NF-κB p65 activity. These findings indicated priming of the NLRP3 inflammasome.

Furthermore, our Western blot analysis showed that the expression of GA50 could significantly increase the expression of NLRP3 (*p* < 0.001), ASC (*p* < 0.001), mature caspase 1 (p20) (*p* < 0.001), pro-IL-1β (*p* < 0.001), mature IL-1β (p17) (*p* < 0.001), pro-IL-18 (*p* < 0.001), and mature IL-18 (*p* < 0.001) levels (Figure 1D). ELISA assays revealed increased secretion of mature IL-1β (*p* < 0.0001) and mature IL-18 (*p* = 0.0002) in the conditional medium (GA-CM) of GA_50_-expressing HMC3 cells (Figure 1E). The above data indicate that intracellularly expressed GA_50_ can induce the activation of NLRP3 inflammasome in HMC3 microglia.

As activation of the NLRP3 inflammasome causes cellular pyroptosis [36], we wanted to assess whether expression of GA_50_ in HMC3 cells would increase cellular pyroptosis. The TUNEL assay showed that the number of positive cells was significantly increased under GA_50_ expression (*p* < 0.001, Figure 1F). We then quantified annexin V/propidium iodide (PI) staining by flow cytometry and found that GA_50_ expression increased the ratio of pyroptosis cells (*p* < 0.001, Figure 1G).

### 3.2. Exocytosis of Mature IL-1 β and IL-18 in GA_50_-Expressing HMC3 Cells Was Inhibited by Treatment with MCC950 Inhibitor of the NLRP3 Inflammasome

**Figure 2:**
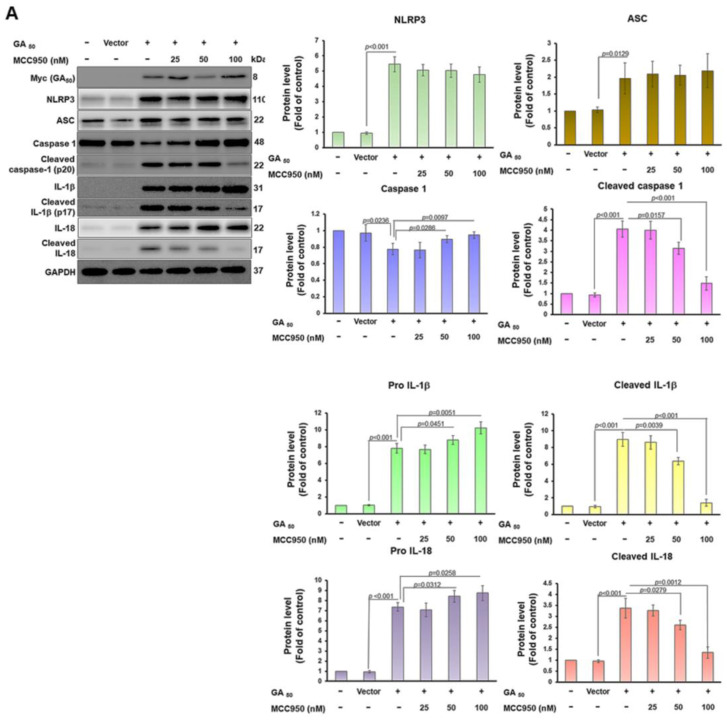

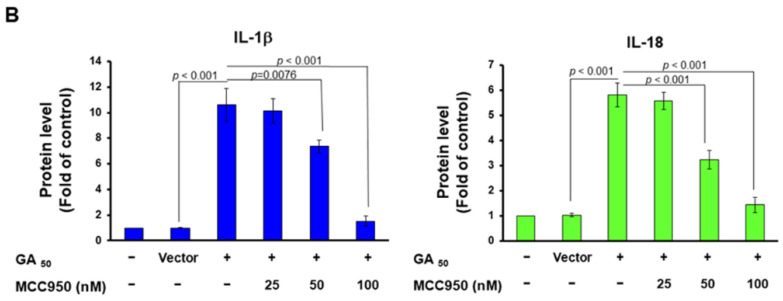
The activity of the NLRP3 inflammasome in GA_50_-expressing HMC3 microglia can be inhibited by MCC950. HMC3 cells that expressed GA_50_ were treated with 0, 25, 50, or 100 nM MCC950 for 24 h. (**A**) Protein levels of GA_50_ (Myc) and NLRP3 inflammasome-associated components in cell lysates detected by Western blot analysis. The internal loading control was GAPDH protein. (**B**) The conditional medium was used to measure the secretion of mature IL-1β and mature IL-18 by ELISA.

To further identity that expression of GA_50_ activates the NLRP3 inflammasome of HMC3 cells, we used an MCC950 inhibitor of the NLPR3 inflammasome. The results showed that after 100 nM MCC950 treatment, the levels of NLRP3 and ASC did not change compared with the untreated group (Figure 2A). Nevertheless, the levels of cleaved caspase 1 (*p*20) (*p* < 0.001), cleaved IL-1β (p17)/pro IL-1β (*p* < 0.001), and cleaved IL-18/pro IL-18 (*p* < 0.001) meaningfully lowered (Figure 2A). Furthermore, we found that exocrine mature IL-1β (*p* < 0.001) and mature IL-18 (*p* < 0.001) were also significantly reduced (Figure 2B). These data confirm that the activity of the NLRP3 inflammasome in HMC3 microglia can be induced by GA-DPR.

### 3.3. GA-DPR Interacts with Sulfide Quinone Oxidoreductase (SQOR) in HMC3 Microglia

**Figure 3:**
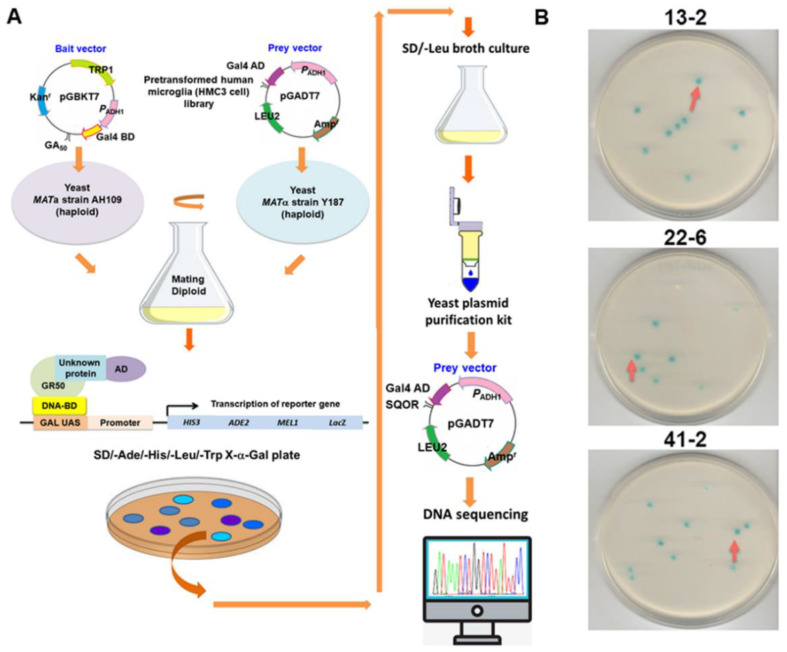

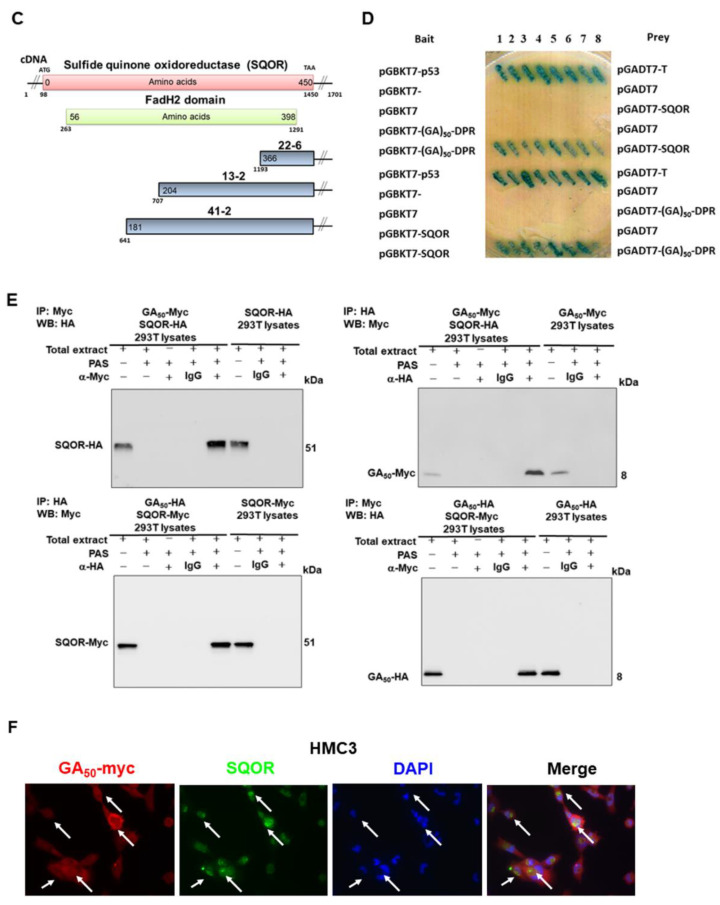
GA-DPR specifically interacts with sulfide quinone oxidoreductase (SQOR) in HMC3 cells. (**A**) Flowchart showing the yeast two-hybrid screening strategy, using GA_50_ as a bait to search for potential interacting proteins in a human HMC3 microglia cDNA library. (**B**) The reporter gene activated by the interaction induces the growth of blue diploid yeast containing positive clones of the *SQOR* cDNA fragment (red arrows). (**C**) Schematic representation of three cDNA clones spanning the coding sequence of the *SQOR* gene (NCBI reference sequence: NP_067022.1). SQOR comprises the FadH2 domain: sulfur reductase. (**D**) The interaction of GA_50_ with full-length SQOR was confirmed using the yeast two-hybrid assay. The positive control group was diploid yeast expressing BD-p53 and AD-T. (**E**) Confirmation of GA_50_ interaction with SQOR using co-immunoprecipitation analysis. 293T cells transfected with only one plasmid were used as a negative control. (**F**) Analysis using immunofluorescence staining confirmed that GA_50_ and SQOR are partially colocalized in the cytoplasm of HMC3 cells (white arrows). DAPI was used to stain the nuclei.

To explore the potential pathways by which GA-DPR activates the NLRP3 inflammasome in microglia, we sought the proteins that might associate with GA_50_ in HMC3 cells. First, we developed a cDNA expression library of HMC3 cells and performed yeast two-hybrid screening using GA_50_ as bait (Figure 3A). Among the positive clones that interacted with GA50, it was confirmed by sequencing that three blue clones included partial cDNA of sulfide quinone oxidoreductase gene (*SQOR*) (NCBI reference sequence: NP_067022.1) (Figure 3B,C).

**Figure 4:**
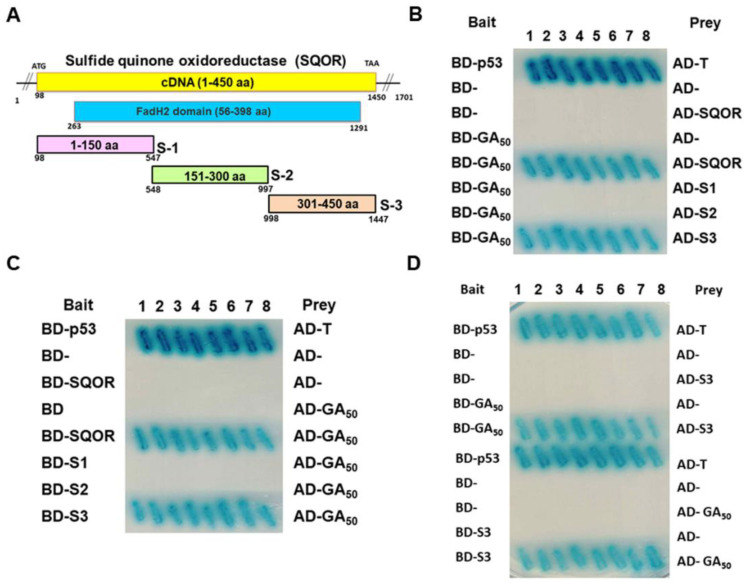
Confirmation of the C-terminus of SQOR as the major interaction region of GA_50_ by the yeast two-hybrid test. (**A**) Schematic showing that SQOR is divided into three fragments for the yeast two-hybrid test. (**B**–**D**) GA_50_ only interacts with the S3 fragment of SQOR in the yeast two-hybrid assay. The positive control was diploid yeast expressing BD-p53 and AD-T.

Next, we implemented a yeast two-hybrid assay of GA_50_ with full-length SQOR and observed the formation of prominent blue colonies (Figure 3D). We then co-expressed GA_50_ and SQOR fused to HA or Myc tags in 293T cells and performed co-immunoprecipitation. The results showed that GA_50_ and SQOR could be identified in the co-immunoprecipitated complex (Figure 3E). Furthermore, we observed that GA_50_ partially co-localized with SQOR in the cytoplasm using immunofluorescence staining (Figure 3F). On the basis of these results, we determined that SQOR could interact with GA_50_. To further confirm the precise interaction region, amino acid sequence of SQOR was divided into three independent fragments, including 1–150 (S1), 151–300 (S2), and 301–450 (S3), and implemented a yeast two-hybrid test with GA_50,_ respectively (Figure 4A). The data showed that GA_50_ primarily associated with the S3 fragment of SQOR (Figure 4B–D).

### 3.4. Downregulation of SQOR Expression in HMC3 Microglia Activates NLRP3 Inflammasome

**Figure 5:**
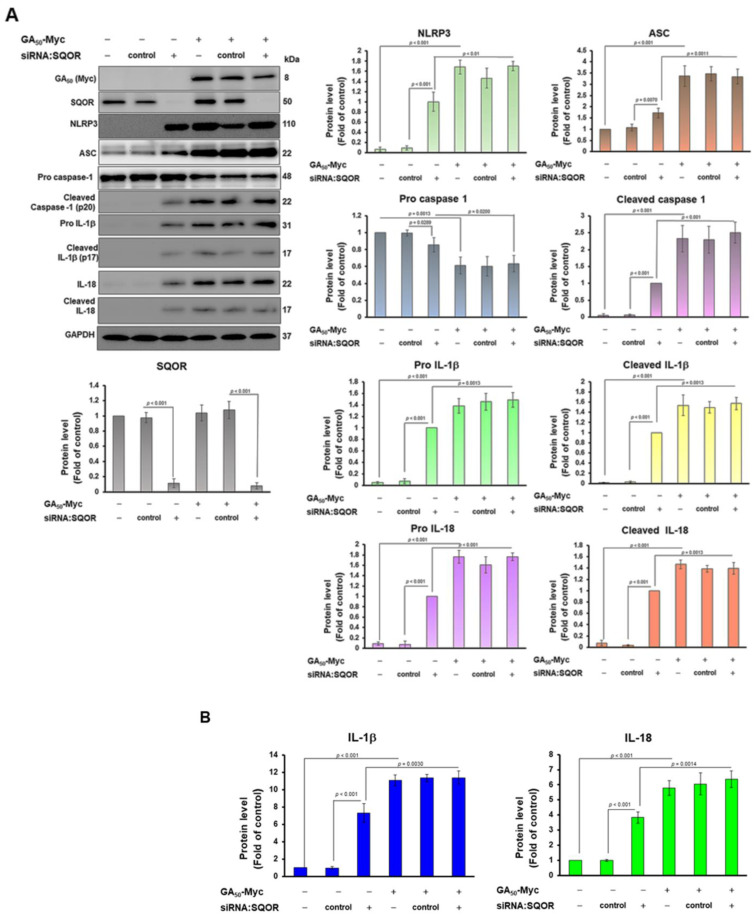
SQOR knockdown in HMC3 cells directly activates NLRP3 inflammasome but fails to enhance the effect of GA_50_. We used siRNA to knockdown the expression of SQOR in non-expressing and GA50-expressing HMC3 cells for 24 h. (**A**) Cell lysates were detected by Western blot for the level of GA50, SQOR, and NLRP3 inflammasome-associated components and quantified using ImageJ software (revision 1.53). GAPDH was used as an internal loading control. (**B**) Exocrine levels of mature IL-1β and mature IL-18 in conditional media were assessed by ELISA.

As SQOR interacts with GA_50_, it is unclear whether this effect mediates NLRP3 inflammasome activity. Therefore, we clarified the role of SQOR expression on NLRP3 inflammasome activation in HMC3 cells. We downregulated the level of SQOR in HMC3 cells using siRNA. Western blot analysis showed that the level of SQOR was significantly decreased in HMC3 cells after *SQOR* siRNA treatment compared with the control siRNA group (*p* < 0.001, Figure 5A). In contrast, in the *SQOR* siRNA group, NLRP3 (*p* < 0.001), ASC (*p* = 0.007), cleaved caspase 1 (*p* < 0.001), pro IL-1β (*p* < 0.001), cleaved IL-1β (*p* < 0.001), pro IL-18 (*p* < 0.001), and cleaved IL-18 (*p* < 0.001) were all significantly increased compared to the control siRNA group (Figure 5A). On the ELISA analysis of conditioned media, the exocytosis of mature IL-1β (*p* < 0.001) and mature IL-18 (*p* < 0.001) was significantly increased in the *SQOR* siRNA group compared with the control siRNA group (Figure 5B). Those results revealed that the inhibition of SQOR expression can activate NLRP3 inflammasome in HMC3 cells.

As a previous experiment confirmed that SQOR knockdown can activate the NLRP3 inflammasome in HMC3 cells, we wanted to confirm whether the inhibition of SQOR expression was the main reason for GA_50_-induced NLRP3 inflammasome activity. We treated GA_50_-expressing HMC3 cells with *SQOR* siRNA for 24 h. Western blotting showed that the expression of SQOR in GA_50_-expressing HMC3 cells was reduced in the *SQOR* siRNA group compared with that in the control siRNA group (*p* < 0.001, Figure 5A). While in the *SQOR* siRNA/GA_50_-expressing group, the levels of NLRP3, ASC, cleaved caspase 1 (p20), pro IL-1β, cleaved IL-1β (p17), pro IL-18, and cleaved IL-18 were not significant changed compared to the control siRNA/GA_50_-expressing group (Figure 5A). In addition, ELISA of conditioned media showed no significant difference in the exocrine levels of mature IL-1β and mature IL-18 in the *SQOR* siRNA/GA_50_-expressing group compared with the control siRNA/GA_50_-expressing group (Figure 5B). These data revealed that SQOR knockdown induced lower activity of the NLRP3 inflammasome than that caused by GA_50_ expression. SQOR knockdown did not significantly enhance GA_50_-induced NLRP3 inflammasome activity. It is indicated that GA_50_ may activate the NLRP3 inflammasome in HMC3 cells partly by binding to inhibit SQOR activity.

### 3.5. GA50 Expression or SQOR Knockdown Increased ROS Generation and Cytoplasmic Escape of Mitochondrial DNA in HMC Cells

**Figure 6:**
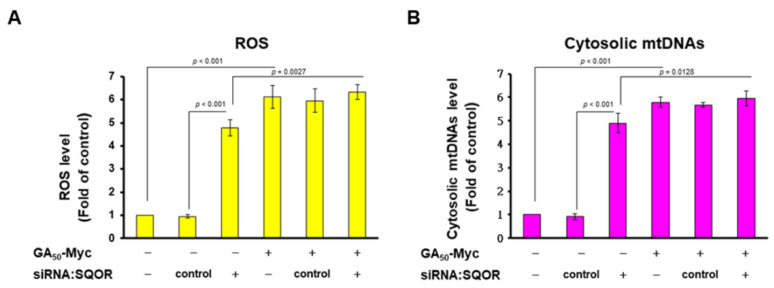
GA_50_ expression or SQOR knockdown increased the generation of ROS and the cytoplasmic escape of mitochondrial DNA in HMC3 cells. (**A**) ROS levels in HMC3 cells with GA_50_ expression or SQOR knockdown were measured by H2DCFDA probe and flow cytometry. (**B**) RT-qPCR was used to analyze the relative changes in the content of mitochondrial ND-1 DNA in the cytoplasm (normalized to ND-1 DNA of total lysates) to determine the degree of cytoplasmic escape of mitochondrial DNA.

Intracellular ROS production and cytoplasmic escape of mitochondrial DNA (mtDNA) due to mitochondrial dysfunction are known to be factors that induce NLRP3 inflammasome activation [40]. SQOR is an important protein involved in the redox reaction and electron transport chain in mitochondria. SQOR deficiency causes mitochondrial dysfunction [41]. Therefore, we further analyzed the effects of SQOR knockdown and GA_50_ expression on ROS production and mtDNA cytoplasmic escape in HMC3 cells. The results showed that the downregulation of SQOR or the expression of GA_50_ significantly increased the generation of ROS (*p* < 0.001, Figure 6A) and the cytoplasmic escape of mtDNA (*p* < 0.001, Figure 6B) in HMC3 cells. The effect of GA_50_ expression on these two phenomena was stronger than that of SQOR knockdown, which is consistent with the difference in the induction of NLRP3 inflammasome activity. While in the *SQOR* siRNA/GA_50_-expressing group, the levels of ROS had no significant changes compared to the control siRNA/GA_50_-expressing group (Figure 6A). In addition, cytoplasmic mtDNA level showed no significant difference in the *SQOR* siRNA/GA_50_-expressing group compared with the control siRNA/GA_50_-expressing group (Figure 6B). These data indicated that GA_50_ may rise ROS and cytoplasmic mtDNA level in HMC3 cells partly by binding to inhibit SQOR activity.

### 3.6. The Interaction between GA50 and SQOR Can Be Inhibited by Irisflorentin in the Yeast Two-Hybrid Model

**Figure 7:**
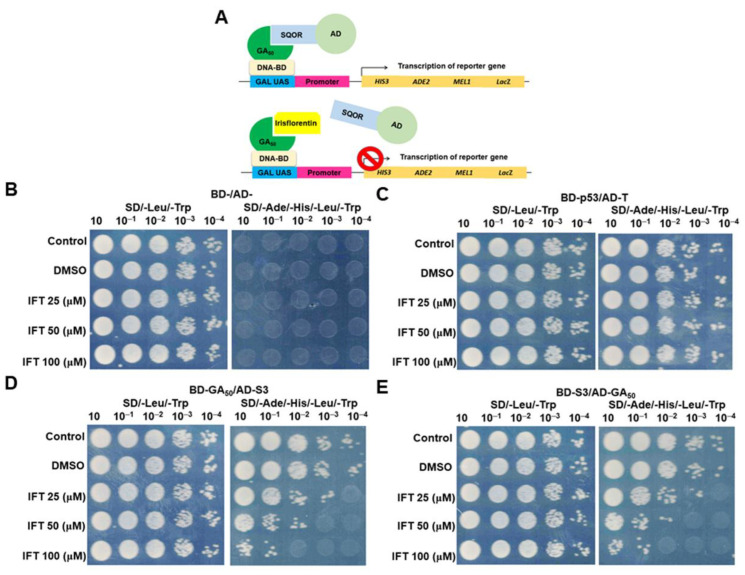

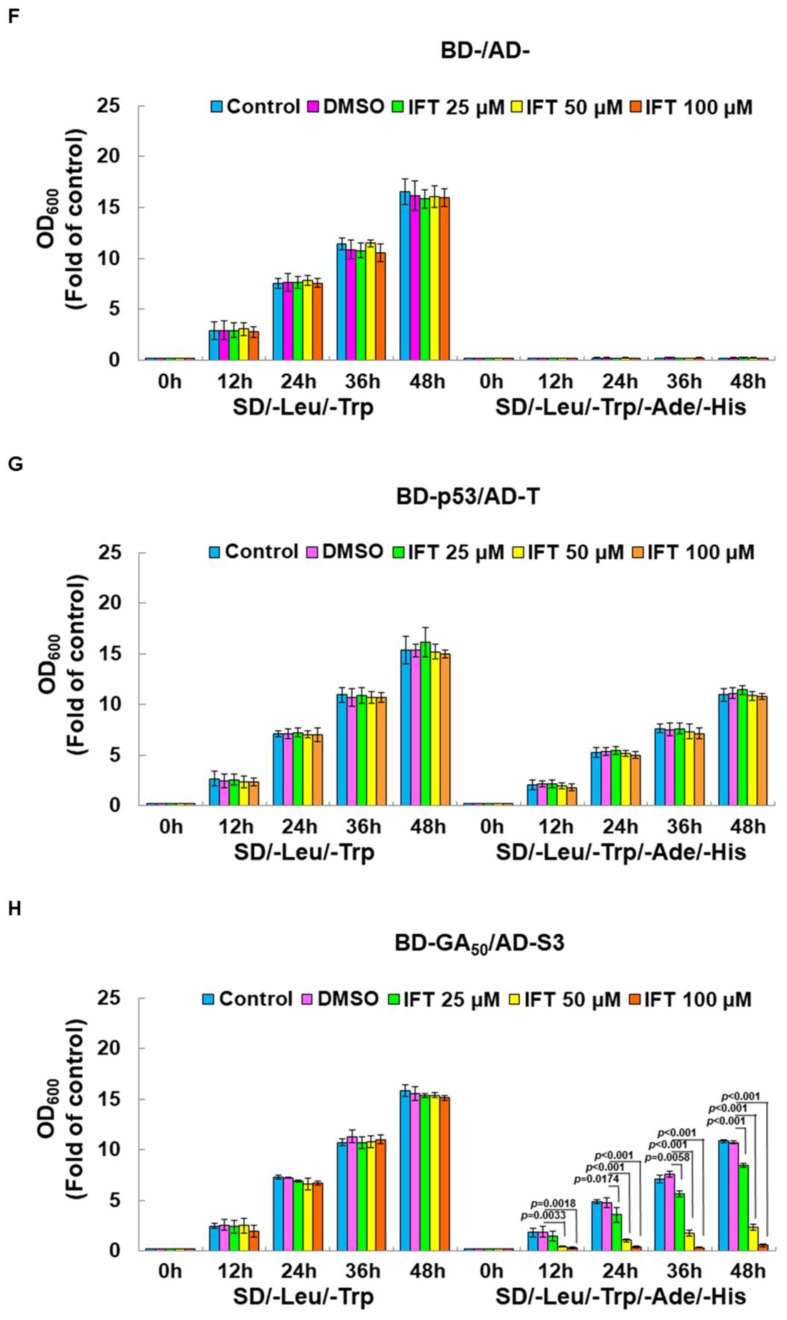

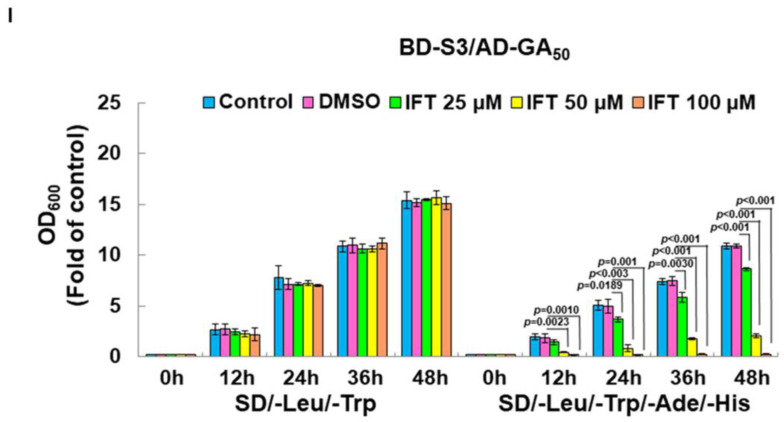
Irisflorentin (IFT) blocks the interaction of GA_50_ and SQOR in a yeast two-hybrid model. (**A**) Schematic showing the strategy based on the yeast two-hybrid principle to screen inhibitors of the interaction between GA_50_ and SQOR. (**B**–**I**) Diploid yeast harboring plasmids encoding BD-/AD-, BD-p53/AD-T, BD-GA_50_/AD-SQOR (S3), or BD-S3/AD-GA_50_ grow to logarithmic phase in non-selective broth (SD/-Leu/-Trp) containing serially diluted IFT. (**B**–**E**) In yeast spot analysis, the amount of diploid yeast in each group was normalized and serially diluted, then spotted on non-selective and selective (SD/-Leu/-Trp/-Ade/-His) culture plates. Spots were incubated at 30°C for 3 days. (**F**–**I**) Diploid yeasts from each group were normalized (OD_600_ = 0.2) and cultured in non-selective and selective broth for 2 days for optical density measurements at 12 h intervals.

As our study found that the main reason for GA_50_ to activate the NLRP3 inflammasome of HMC3 cells is its interaction with SQOR, we hope to establish a feasible method to reverse the pathological progression of C9-ALS by disrupting the interaction between GA_50_ and SQOR, such as finding small molecule inhibitors. We used a yeast two-hybrid-based growth assay [42] to screen dozens of small chemical molecules in our laboratory (Figure 7A). Screening results revealed that irisflorentin (IFT) has this property. The results of yeast spot assay and quantification of the optical density of the yeast culture showed that the growth of BD-/AD-diploid yeast on the non-selective(SD/-Leu/-Trp) plate (or broth) was not significantly affected after IFT treatment below 100 μM. However, it failed to grow on selective (SD/-Ade/-His/-Leu/-Trp) plates (or broth) as predicted (Figure 7B,F). The growth of diploid yeast of BD-P53/AD-T in non-selective or selective culture plates (or broth) under 100 μM IFT treatment was not affected, but growth was slightly slower on selective plates (or broth) (Figure 7C,G). The above observations indicated that IFT treatment below 100 μM was not significantly toxic to yeast. Next, we found that diploid yeast of BD-GA_50_/AD-SQOR(S3) grew unaffected on SD/-Leu/-Trp plates (broth) by 100 μM IFT treatment. However, growth in selective culture plates (or broth) showed a IFT dose-dependent decrease (*p* < 0.001, 100 μM IFT, 48 h) (Figure 7D,H). This indicates that the interaction between GA_50_ and SQOR is blocked by IFT treatment. Diploid yeast experiments with S3/AD-GA_50_ also showed the same results (*p* < 0.001, 100 μM IFT, 48 h) (Figure 7E,I).

### 3.7. Irisflorentin (IFT) Reverses GA_50_-Caused Activity of NLRP3-Inflammasome in HMC3 Cells

**Figure 8:**
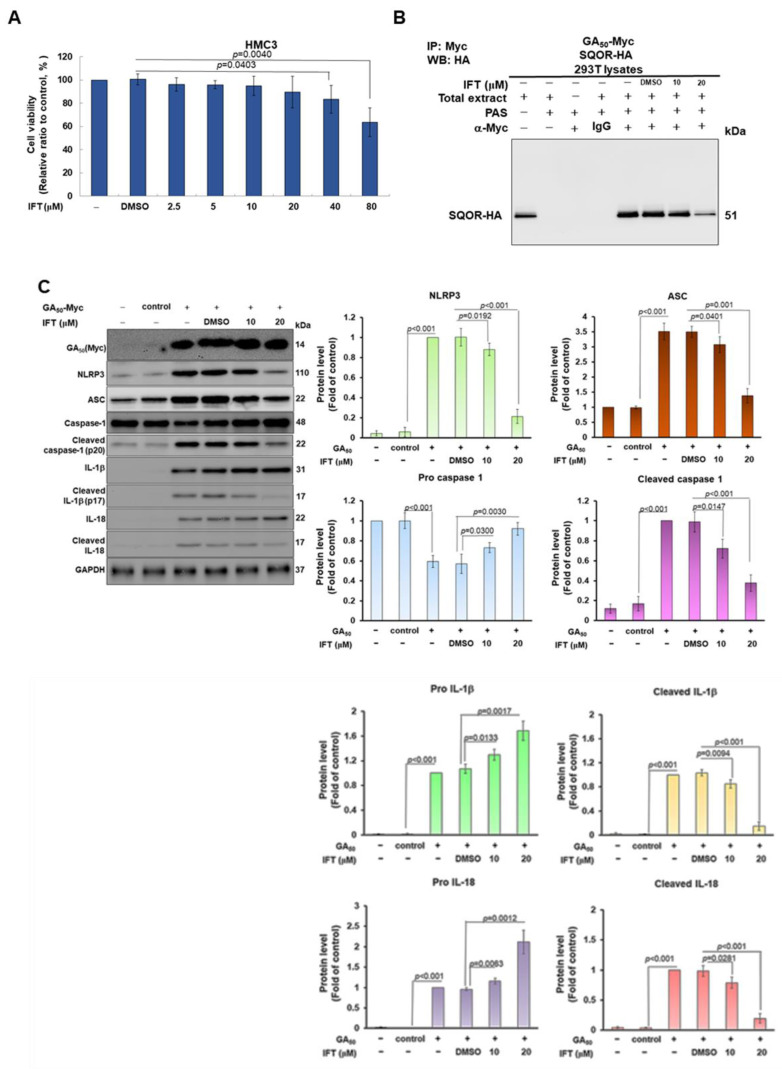

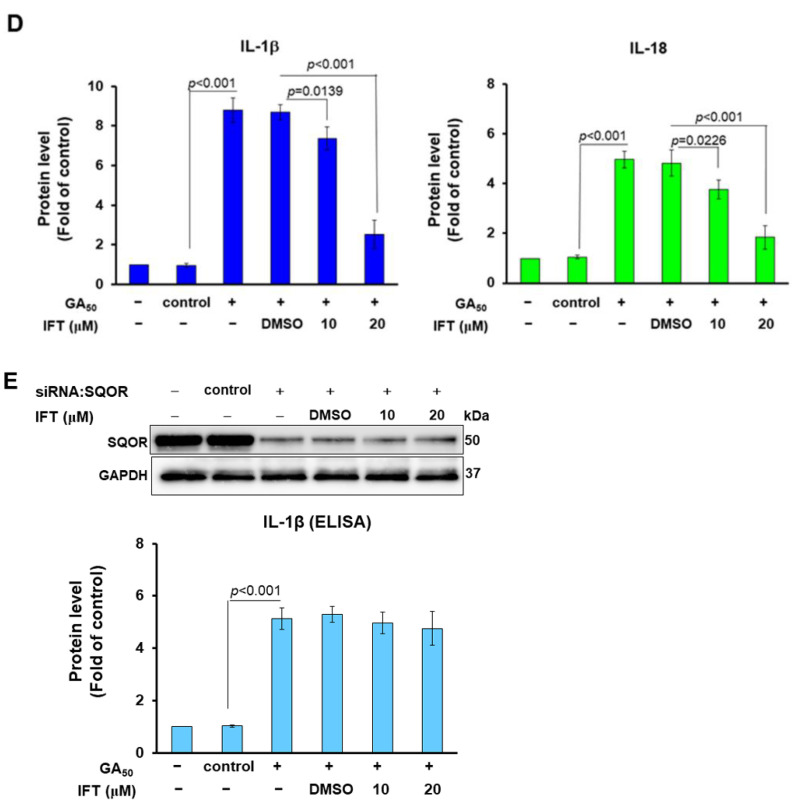
Irisflorentin (IFT) reverses NLRP3 inflammasome activity in GA_50_-expressing HMC3 microglia. (**A**) Cell viability was determined by MTT assay. HMC3 cells were analyzed after treatment with DMSO, 2.5, 5, 10, 20, 40, or 80 μM IFT for 24 h. (**B**) IFT inhibits the interaction of GA_50_ and SQOR in a co-immunoprecipitation assay of 293T cells expressing GA_50_-Myc and SQOR-HA. (**C**) Expression of GA_50_ (Myc) and NLRP3 inflammasome-associated components in cell lysates was quantified by Western blotting and ImageJ software (version 1.53). The internal loading control protein is GAPDH. (**D**) The exocrine levels of mature IL-1β and mature IL-18 in conditioned media were determined by ELISA. (**E**) Western blot analysis of the expression of SQOR and NLRP3 inflammasome-associated components in SQOR-knockdown HMC3 cells (**top** panel). GAPDH served as an internal loading control. Mature IL-1β activity in the conditional medium was detected by ELISA (**bottom** panel).

The previous experiment showed that IFT can block the interaction between GA_50_ and SQOR in the yeast two-hybrid model; therefore, we further assessed whether IFT can inhibit the activity of the NLRP3 inflammasome induced by GA_50_ in HMC3 cells. First, to prevent cytotoxicity from the IFT concentrations used for treatment, we performed MTT assays. The results showed that the survival of HMC3 cells was not significantly affected after 24 h of IFT treatment below 20 μM (Figure 8A). We also found that the co-immunoprecipitation interaction of GA_50_ and SQOR in 293T cells was accompanied by a concentration-dependent reduction in IFT at IFT-treated concentrations below 20 μM (Figure 8B). Moreover, GA_50_-caused activity of the NLRP3 inflammasome in HMC3 cells was IFT concentration-dependently decreased after 24 h treatment. Quantified by Western blotting, the levels of NLRP3 (*p* < 0.001), ASC (*p* < 0.001), cleaved caspase 1 (p20) (*p* < 0.001), cleaved IL-1β (p17) (*p* < 0.001), and cleaved IL-18 (*p* < 0.001) were significantly diminished (Figure 8C). ELISA for the determination of the conditioned medium also showed that the exocrine levels of mature IL-1β (*p* < 0.001) and mature IL-18 (*p* < 0.001) in HMC3 cells expressing GA_50_, compared with the DMSO group, in the IFT group (20 μM) were also significantly reduced (*p* < 0.001 Figure 8D). To more precisely determine that the function of IFT is to inhibit interaction of GA_50_ and SQOR rather than NLRP3 inflammasome activity, we treated HMC3 cells of SQOR-knockdown with IFT. The results revealed that the upregulated NLRP3 inflammasome activity was not significantly reduced (Figure 8E). The above data indicated that IFT could specifically reverse the activity of GA_50_-induced NLRP3 inflammasome in microglia by blocking the interaction between GA_50_ and SQOR.

## 4. Discussion

C9-ALS patients have an augmented tendency to develop autoimmune diseases due to the continuous production of uncontrolled inflammatory cytokines. This indicates that *C9orf72* mutation directly or indirectly affects some inflammatory events [43]. Microglia are the most important immune cells in the brain and play an important role in maintaining the health of neurons and homeostasis of niches [44]. Activation of the NLRP3 inflammasome in microglia is known to be an important pathological factor in ALS [45]. Priming and activation are two essential steps for activating the canonical NLRP3 inflammasome [46]. Priming induces NF-κB transcriptional activity and are involved in the expression of NLRP3, IL-1β, and IL-18. Next, various factors including ROS promote NLRP3 oligomerization and recruit CARD-containing adapter protein apoptosis-associated speck-like protein (ASC), which then binds procaspase-1 to form a complex called the inflammasome. Finally, pro-caspase-1 self-cleavage and activation convert pro-IL-1β and pro-IL-18 to active mature forms and releases them. At the same time, it also cleaves the N-terminal fragment of gasdermin-D (GSDMD) to form a cleavage hole, which promotes pyroptosis. This phase is called activation [47]. Mature IL-1β and IL-18 mediate inflammatory responses to cause damage and death of neighboring neurons [48]. Observations of ALS patients and *TDP-43* and *SOD1* mutant mice showed NLRP3 activation, increases in IL-18, and cleavages of GSDMD in microglial cells distributed in the spinal cord and motor cortex of the brain [49,50,51].

In this study, we found that the endogenous expression of C9-ALS associated GA-DPR (GA_50_) induced the priming and activation of the NLRP3 inflammasome in the HMC3 human microglial cell line. MCC950 (NLRP3 inhibitor) treatment confirmed the role of GA-DPR in NLRP3 inflammasome activation. Some abnormal protein aggregation associated with neurodegenerative diseases, such as amyloid beta peptide (Aβ) in Alzheimer’s disease [52], α-synuclein in Parkinson’s disease [53], and SOD1 and TDP-43 [51] in ALS, are known to induce the activity of the NLRP3 inflammasome in a mouse model. Recently, Shu et al. also reported that GA-DPR can activate the NLRP3 inflammasome in mouse brain microglial cells, but they did not explore the mechanism of NLRP3 inflammasome activation. They found that aberrant NLRP3 inflammasome activity enhances ADAM10-mediated TREM2 cleavage, leading to the inhibition of GA-DPR phagocytosis [54]. In addition, we found that GA_50_ also induces apoptosis in HMC3 cells in addition to the pyroptosis induced by NLRP3 inflammasome activation. This may be because caspase-1 can activate the apoptosis process by cleaving and activating caspase-3 [55].

To explore the possible mechanism of GA-DPR activating the NLRP3 inflammasome, we used ELISA to detect GA-DPR (Myc) in the conditional medium. The results showed a weak signal, indicating that a small amount of GA-DPR was transported out of the cell and may bind to a specific receptor on the cell surface as a DAMP to induce priming of the NLRP3 inflammasome. In addition, we confirmed that GA-DPR interacts with sulfide quinone oxidoreductase (SQOR) by yeast two-hybrid screening. The interaction region is located at the C-terminal fragment of SQOR (amino acid sequence 301–450). This region contains a part of the enzyme active region. Up to now, there are no detailed reports about the function of SQOR. It may function in mitochondria to catalyze the conversion of sulfide to persulfides, thereby decreasing the toxic concentrations of sulfide [56,57]. The low level of SQOR in the brain and spinal cord in most mammals results in a limited ability to catabolize sulfide in these regions. Therefore, these regions are particularly sensitive to the toxicity of sulfide accumulation [58]. Moreover, under physiological conditions, SQOR activity promotes mitochondrial ATP synthesis. This is achieved by sulfide oxidation, which donates electrons to complex III of the mitochondrial electron transport chain (ETC) via coenzyme Q (CoQ), or by generating persulfides that can serve as electron acceptors of ETC [59]. The study showed that increasing the expression of SQOR improves ischemic brain injury [58]. Furthermore, the downregulation of SQOR in HeLa cells increased ROS in the cells [60]. Studies have shown that oxidative stress disrupts the mitochondrial membrane potential, leading to depolarization. In addition to affecting the normal function of mitochondria, depolarization can also induce the opening of mitochondrial permeability transition pore (MPTP), which allows the release of mitochondrial ROS (mtROS), DNA (mtDNA), or intermembrane space proteins into the cytoplasm [61]. Both mtROS and mtDNA are active factors that induce NLRP3 inflammasome [39]. In particular, cytosolic, oxidized mtDNA can activate the inflammasome by binding to the inflammasome sensors AIM2 and NL RP3 [62].

As the relationship between SQOR and NLRP3 inflammasome is not clear, we use siRNA to down-regulate SQOR in microglial cells to measure the change in NLRP3 inflammasome activity. The results showed that SQOR knockdown could induce NLRP3 inflammasome activity as significantly as GA-DPR expression. Moreover, we found that both GA_50_ expression and SQOR knockdown in HMC3 cells significantly induced ROS production and cytoplasmic mtDNA escape. However, HMC3 cells with GA_50_ expression and SQOR downregulation did not enhance NLRP3 inflammasome activity compared with GA_50_-expressing cells. According to the above results, the expression of GA_50_ in microglial cells may partly cause the increase in mitochondrial ROS and the cytoplasmic escape of mtDNA through the inhibition of SQOR activity, thereby activating the NLRP3 inflammasome.

Another possible effect of GA_50_ on the inhibition of SQOR function is the accumulation of SQOR substrates hydrogen sulfide (H_2_S) in cells [59]. H_2_S is an environmental toxin and an endogenous gasotransmitter (or neurotransmitter). It is an essential molecule that not only acts as a vasodilator, cytoprotectant, antioxidant, and anti-inflammatory agent but also acts as a poison [63]. It is generally believed that H_2_S has beneficial effects at physiological concentrations (nM to low-μM) but may lead to harmful effects at higher concentrations [64]. A study showed high H_2_S levels in the cerebrospinal fluid and nerve tissue of SOD1 mutant ALS mice and sporadic ALS patients [65]. This H_2_S mainly comes from glial cells [65]. In addition, the study found that a higher H_2_S concentration increases the intracellular calcium ion concentration of motor neurons, inhibits mitochondrial respiration, and reduces ATP production. Higher H_2_S levels also enhance pathways related to oxidative stress and cell death [65]. In the analysis of the NLRP3 inflammasome in microglial cells, some studies have shown that H_2_S treatment can inhibit the activity of the NLRP3 inflammasome [66,67]. However, some studies have also shown that H2S exposure can induce the secretion of NLRP3 inflammasome-dependent IL-1β and IL-18 in human mononuclear leukocytes [68] and broiler thymocytes [69]. This effect may be because H_2_S activates the NLRP3 inflammasome through the TLR-7/MyD 88/NF-κB pathway [69]. These differential effects on NLRP3 inflammasome activity may be related to the concentration of H_2_S treatment, cell types, or animal models. Although we did not measure the amount of H2S in HMC3 cells with or without expression of GA_50_ in this study, we confirmed that part of the active region of the SQOR enzyme was bound by GA_50_ and blocked, so it is likely to increase the amount of H_2_S in HMC3 cells to the level of toxicity and induce NLRP3 inflammasome activity.

Finally, we found that irisflorentin (IFT) can inhibit the interaction between GA_50_ and SQOR by using yeast two-hybrid-based growth assay and co-immunoprecipitation technique. Likewise, IFT treatment reduced GA_50_-induced NLRP3 inflammasome activity. However, irisflorentin could not inhibit the activation of NLRP3 inflammasome caused by the downregulation of SQOR. It was confirmed that IFT specifically inhibited the interaction between GA50 and SQOR to prevent the activation of the NLRP3 inflammasome. IFT is isolated from the root of *Belamcanda chinensis* (L.) DC, which has various biological activities. [70]. Studies have shown that IFT can enhance the expression of antioxidant enzymes and improve keratinocyte apoptosis and collagen degradation caused by ultraviolet-B-induced ROS [71]. IFT has also been shown to ameliorate 6-OHDA-induced dopaminergic neuronal degeneration and α-synuclein accumulation in Parkinson’s disease models [72]. Moreover, IFT can inhibit allergic contact hypersensitivity by modifying the properties of mouse bone marrow dendritic cells [73] and can improve the LPS-induced inflammatory response of RAW 264.7 macrophages [74]. Therefore, in addition to diminishing the activity of the NLRP3 inflammasome induced by GA_50_, IFT may also have neuroprotective activities in regulating cellular anti-oxidation, anti-inflammation, and clearing amyloid in C9-ALS patients. Several studies have shown that the inhibitor of the NLRP3 inflammasome (MCC950) can effectively reverse the motor deficits in ALS-containing GA-DPR-expressing mice caused by the activation of the NLRP3 inflammasome [54]. However, this non-specific broad inhibition of NLRP3 inflammasome activity may disrupt innate immune mechanisms and increase the chances of infection in patients. Therefore, for C9-ALS, the therapeutic strategy of IFT can avoid this risk.

Taken together, the results of this study provide a novel explanation for the establishment of C9-ALS. GA-DPR may cause the generation of ROS and cytoplasmic escape of mtDNA in microglial cells by binding and regulating the activity of SQOR. Finally, the activation of the NLRP3 inflammasome affects the survival and function of surrounding neurons. In addition, we found that IFT is a specific inhibitor of this abnormal interaction and has the potential to be developed as an anti-C9-ALS drug.

## Data Availability

The data presented in this study are available on request from the corresponding author.
